# Supplementary material for: Real-World Experiences Using Atezolizumab + Bevacizumab for the Treatment of Unresectable Hepatocellular Carcinoma: A Multicenter Study
Source: Cancers (Basel). 2025 May 29;17(11):1814. doi: 10.3390/cancers17111814 (PMC12153728; doi:10.3390/cancers17111814)
Supplement: Supplementary file 1 [file cancers-17-01814-s001.zip › cancers-3624924-supplementary.pdf]

Real-World Experiences Using Atezolizumab + Bevacizumab for the Treatment of Unresectable Hepatocellular Carcinoma: A Multicenter Study

Supplementary Materials

Supplementary Table S1. Demographic characteristics among select subgroups of uHCC patients receiving A+B as 1L treatment 2

Supplementary Table S2. Clinical and disease characteristics among select subgroups of uHCC patients receiving A+B as 1L treatment 3

Supplementary Table S3. Use of EGD during the baseline period among patients with uHCC receiving A+B as 1L treatment 4

Supplementary Table S4. HCC-related healthcare resource utilization in patients with uHCC receiving A+B as 1L treatment, during the first year of treatment with A+B 5

Supplementary Table S1. Demographic characteristics among select subgroups of uHCC patients receiving A+B as 1L treatment.

	"Trial-like" characteristics			CP class			Race/ethnicity					
	Yes N=194	No N=106	*P*-value[1]	CP A N=219	CP B N=79	*P*-value[1]	White (non-Hispanic) N=195	Black (non-Hispanic) N=35	Asian (non-Hispanic)[2] N=21	Hispanic or Latino N=39	Unknown N=10	*P*-value[1]
**Demographic Characteristics**												
Patient age at initiation of 1L A+B[2] (Mean ± SD; years)	67.5 ± 9.6	67.2 ± 8.7	0.82	67.6 ± 9.3	66.9 ± 9.1	0.56	68.1 ± 8.9	65.9 ± 9.6	66.4 ± 11.3	66.3 ± 9.9	65.5 ± 8.1	0.54
Male, n (%)	149 (76.8%)	89 (84.0%)	0.19	169 (77.2%)	69 (87.3%)	0.08	157 (80.5%)	26 (74.3%)	19 (90.5%)	29 (74.4%)	7 (70.0%)	0.45
**Race[3,4], n (%)**												
White	151 (77.8%)	85 (80.2%)	0.74	171 (78.1%)	63 (79.7%)	0.88	195 (100.0%)	0 (0.0%)	0 (0.0%)	36 (92.3%)	5 (50.0%)	< 0.001 *
Black or African American	24 (12.4%)	11 (10.4%)	0.74	28 (12.8%)	7 (8.9%)	0.47	0 (0.0%)	35 (100.0%)	0 (0.0%)	0 (0.0%)	0 (0.0%)	< 0.001 *
Asian	12 (6.2%)	6 (5.7%)	1.00	13 (5.9%)	5 (6.3%)	1.00	0 (0.0%)	0 (0.0%)	18 (85.7%)	0 (0.0%)	0 (0.0%)	< 0.001 *
Others[5]	3 (1.5%)	2 (1.9%)	1.00	3 (1.4%)	2 (2.5%)	0.61	0 (0.0%)	0 (0.0%)	3 (14.3%)	2 (5.1%)	0 (0.0%)	< 0.001 *
**Ethnicity[4], n (%)**												
Hispanic or Latino	23 (11.9%)	16 (15.1%)	0.54	24 (11.0%)	15 (19.0%)	0.11	0 (0.0%)	0 (0.0%)	0 (0.0%)	39 (100.0%)	0 (0.0%)	< 0.001 *
Not Hispanic or Latino	167 (86.1%)	88 (83.0%)	0.59	190 (86.8%)	63 (79.7%)	0.19	195 (100.0%)	35 (100.0%)	21 (100.0%)	0 (0.0%)	4 (40.0%)	< 0.001 *
**Education, n (%)**												
High school diploma or less	32 (16.5%)	17 (16.0%)	1.00	35 (16.0%)	14 (17.7%)	0.86	38 (19.5%)	2 (5.7%)	3 (14.3%)	4 (10.3%)	2 (20.0%)	0.22
Some college or associate's degree	27 (13.9%)	15 (14.2%)	1.00	30 (13.7%)	12 (15.2%)	0.89	36 (18.5%)	2 (5.7%)	4 (19.0%)	0 (0.0%)	0 (0.0%)	< 0.01 *
College graduate, bachelor's degree, or advanced degree	40 (20.6%)	15 (14.2%)	0.22	44 (20.1%)	11 (13.9%)	0.30	48 (24.6%)	1 (2.9%)	4 (19.0%)	2 (5.1%)	0 (0.0%)	< 0.001 *
Unknown	95 (49.0%)	59 (55.7%)	0.32	110 (50.2%)	42 (53.2%)	0.75	73 (37.4%)	30 (85.7%)	10 (47.6%)	33 (84.6%)	8 (80.0%)	< 0.001 *
**Employment status**												
Employed[6]	35 (18.0%)	16 (15.1%)	0.63	37 (16.9%)	14 (17.7%)	1.00	36 (18.5%)	3 (8.6%)	5 (23.8%)	6 (15.4%)	1 (10.0%)	0.56
Unemployed[7]	14 (7.2%)	8 (7.5%)	1.00	16 (7.3%)	6 (7.6%)	1.00	14 (7.2%)	1 (2.9%)	4 (19.0%)	3 (7.7%)	0 (0.0%)	0.26
Retired	103 (53.1%)	54 (50.9%)	0.81	118 (53.9%)	39 (49.4%)	0.58	112 (57.4%)	15 (42.9%)	9 (42.9%)	17 (43.6%)	4 (40.0%)	0.21
On disability[8]	29 (14.9%)	8 (7.5%)	0.09	30 (13.7%)	7 (8.9%)	0.36	19 (9.7%)	8 (22.9%)	1 (4.8%)	8 (20.5%)	1 (10.0%)	0.08
Unknown	24 (12.4%)	23 (21.7%)	0.05	30 (13.7%)	15 (19.0%)	0.35	26 (13.3%)	8 (22.9%)	3 (14.3%)	6 (15.4%)	4 (40.0%)	0.15
**Insurance type[3], n (%)**												
Commercial/private insurance	135 (69.6%)	69 (65.1%)	0.50	150 (68.5%)	52 (65.8%)	0.77	141 (72.3%)	25 (71.4%)	13 (61.9%)	19 (48.7%)	6 (60.0%)	0.06
Medicare	56 (28.9%)	39 (36.8%)	0.20	64 (29.2%)	31 (39.2%)	0.13	68 (34.9%)	6 (17.1%)	6 (28.6%)	10 (25.6%)	5 (50.0%)	0.15
Medicaid	16 (8.2%)	5 (4.7%)	0.36	20 (9.1%)	1 (1.3%)	< 0.05 *	7 (3.6%)	2 (5.7%)	2 (9.5%)	9 (23.1%)	1 (10.0%)	< 0.01 *
Other	7 (3.6%)	4 (3.8%)	1.00	10 (4.6%)	1 (1.3%)	0.30	7 (3.6%)	1 (2.9%)	2 (9.5%)	1 (2.6%)	0 (0.0%)	0.60
Unknown	6 (3.1%)	5 (4.7%)	0.53	7 (3.2%)	4 (5.1%)	0.49	6 (3.1%)	1 (2.9%)	2 (9.5%)	1 (2.6%)	1 (10.0%)	0.25

Abbreviations – 1L: first-line; A+B: atezolizumab plus bevacizumab; CP: Child-Pugh; SD: standard deviation; uHCC: unresectable hepatocellular carcinoma.

Notes:

1. Statistical comparisons between subgroups were performed using chi-squared tests for categorical variables. Where expected counts ≤ 10, Fisher's exact tests were used instead of chi-squared tests. *P*-value < 0.05 was considered statistically significant and is denoted with an asterisk (*).
2. For patient privacy, the exact age of patients aged ≥ 90 years was not collected. Age at initiation of 1L A+B treatment for these patients was set to 90 years by default in the calculation of summary statistics.
3. Multiple responses were allowed for this question and percentages may add up to more than 100%.
4. Percentages may not add up to 100% because a small proportion of patients with unknown information has not been reported in the table.
5. Other race categories were American Indian or Alaska Native, or Hawaiian.
6. Employed represented patients who were self-employed, employed full-time, or employed part-time.
7. Unemployed represented patients who reported to be unemployed as well as homemakers.
8. "On disability" categories were allowed to be selected together with all other categories except "Unknown".

Supplementary Table S2. Clinical and disease characteristics among select subgroups of uHCC patients receiving A+B as 1L treatment.

	"Trial-like" characteristics			CP class			Race/ethnicity					
	Yes N=194	No N=106	*P*-value[1]	CP A N=219	CP B N=79	*P*-value[1]	White (non-Hispanic) N=195	Black (non-Hispanic) N=35	Asian (non-Hispanic)[2] N=21	Hispanic or Latino N=39	Unknown N=10	*P*-value[1]
**Primary HCC etiology, n (%)**												
Viral etiology[3]	90 (46.4%)	42 (39.6%)	0.31	99 (45.2%)	32 (40.5%)	0.56	67 (34.4%)	23 (65.7%)	15 (71.4%)	21 (53.8%)	6 (60.0%)	< 0.001 *
Non-viral etiology[3]	57 (29.4%)	51 (48.1%)	< 0.01 *	68 (31.1%)	39 (49.4%)	< 0.01 *	76 (39.0%)	8 (22.9%)	3 (14.3%)	17 (43.6%)	4 (40.0%)	0.06
Other etiology[4]	24 (12.4%)	11 (10.4%)	0.74	28 (12.8%)	7 (8.9%)	0.47	29 (14.9%)	4 (11.4%)	1 (4.8%)	1 (2.6%)	0 (0.0%)	0.14
Unknown etiology	23 (11.9%)	2 (1.9%)	< 0.01 *	24 (11.0%)	1 (1.3%)	< 0.01 *	23 (11.8%)	0 (0.0%)	2 (9.5%)	0 (0.0%)	0 (0.0%)	< 0.05 *
**CP class, within 90 days of initiation of 1L A+B treatment, n (%)**												
A (CP score = 5-6 points)	194 (100.0%)	25 (23.6%)	< 0.001 *	219 (100.0%)	0 (0.0%)	< 0.001 *	144 (73.8%)	28 (80.0%)	16 (76.2%)	24 (61.5%)	7 (70.0%)	0.45
B (CP score = 7-9 points)	0 (0.0%)	79 (74.5%)	< 0.001 *	0 (0.0%)	79 (100.0%)	< 0.001 *	49 (25.1%)	7 (20.0%)	5 (23.8%)	15 (38.5%)	3 (30.0%)	0.42
C (CP score = 10-15 points)	0 (0.0%)	2 (1.9%)	0.12	0 (0.0%)	0 (0.0%)	-	2 (1.0%)	0 (0.0%)	0 (0.0%)	0 (0.0%)	0 (0.0%)	1.00
**BCLC stage[5], within 90 days of initiation of 1L A+B treatment, n (%)**												
A: Early	8 (4.1%)	0 (0.0%)	0.05	8 (3.7%)	0 (0.0%)	0.12	4 (2.1%)	1 (2.9%)	0 (0.0%)	2 (5.1%)	1 (10.0%)	0.25
B: Intermediate	25 (12.9%)	16 (15.1%)	0.72	28 (12.8%)	13 (16.5%)	0.53	32 (16.4%)	3 (8.6%)	1 (4.8%)	4 (10.3%)	1 (10.0%)	0.52
C: Advanced	161 (83.0%)	86 (81.1%)	0.81	182 (83.1%)	65 (82.3%)	1.00	156 (80.0%)	30 (85.7%)	20 (95.2%)	33 (84.6%)	8 (80.0%)	0.48
D: End-stage	0 (0.0%)	3 (2.8%)	< 0.05 *	0 (0.0%)	1 (1.3%)	0.27	2 (1.0%)	1 (2.9%)	0 (0.0%)	0 (0.0%)	0 (0.0%)	0.56
**ECOG PS[5], within 90 days of initiation of 1L A+B treatment, n (%)**												
Grade 0: Fully active	85 (43.8%)	25 (23.6%)	< 0.001 *	87 (39.7%)	23 (29.1%)	0.12	70 (35.9%)	10 (28.6%)	8 (38.1%)	19 (48.7%)	3 (30.0%)	0.46
Grade 1: Restricted	109 (56.2%)	38 (35.8%)	< 0.01 *	110 (50.2%)	35 (44.3%)	0.44	100 (51.3%)	19 (54.3%)	11 (52.4%)	13 (33.3%)	4 (40.0%)	0.28
Grade 2: Ambulatory	0 (0.0%)	34 (32.1%)	< 0.001 *	18 (8.2%)	16 (20.3%)	< 0.01 *	21 (10.8%)	2 (5.7%)	2 (9.5%)	6 (15.4%)	3 (30.0%)	0.25
Grade 3: Confined to bed or chair	0 (0.0%)	1 (0.9%)	0.35	0 (0.0%)	1 (1.3%)	0.27	0 (0.0%)	1 (2.9%)	0 (0.0%)	0 (0.0%)	0 (0.0%)	0.22
**HCC tumor invasion[6], within 90 days of initiation of 1L A+B treatment, n (%)**												
Extrahepatic spread	81 (41.8%)	39 (36.8%)	0.47	87 (39.7%)	32 (40.5%)	1.00	68 (34.9%)	17 (48.6%)	13 (61.9%)	18 (46.2%)	4 (40.0%)	0.09
Vp4 portal vein invasion	38 (19.6%)	38 (35.8%)	< 0.01 *	49 (22.4%)	26 (32.9%)	0.09	55 (28.2%)	4 (11.4%)	8 (38.1%)	8 (20.5%)	1 (10.0%)	0.09
Tumor invasion >50% of liver	36 (18.6%)	21 (19.8%)	0.91	40 (18.3%)	17 (21.5%)	0.64	41 (21.0%)	4 (11.4%)	4 (19.0%)	7 (17.9%)	1 (10.0%)	0.74
Bile duct invasion	1 (0.5%)	12 (11.3%)	< 0.001 *	4 (1.8%)	9 (11.4%)	< 0.01 *	10 (5.1%)	1 (2.9%)	1 (4.8%)	1 (2.6%)	0 (0.0%)	0.97
None of the above	76 (39.2%)	32 (30.2%)	0.15	84 (38.4%)	24 (30.4%)	0.26	72 (36.9%)	12 (34.3%)	4 (19.0%)	14 (35.9%)	6 (60.0%)	0.27
**ALBI grade[5,7], within 90 days of initiation of 1L A+B treatment, n (%)**												
1	87 (44.8%)	11 (10.4%)	< 0.001 *	95 (43.4%)	3 (3.8%)	< 0.001 *	74 (37.9%)	11 (31.4%)	6 (28.6%)	3 (7.7%)	4 (40.0%)	< 0.01 *
2	107 (55.2%)	66 (62.3%)	0.29	121 (55.3%)	51 (64.6%)	0.19	104 (53.3%)	22 (62.9%)	15 (71.4%)	28 (71.8%)	4 (40.0%)	0.09
2A	60 (56.1%)	26 (39.4%)	< 0.05 *	68 (56.2%)	18 (35.3%)	< 0.05 *	53 (51.0%)	8 (36.4%)	9 (60.0%)	15 (53.6%)	1 (25.0%)	0.51
2B	47 (43.9%)	40 (60.6%)	< 0.05 *	53 (43.8%)	33 (64.7%)	< 0.05 *	51 (49.0%)	14 (63.6%)	6 (40.0%)	13 (46.4%)	3 (75.0%)	0.51
3	0 (0.0%)	27 (25.5%)	< 0.001 *	2 (0.9%)	25 (31.6%)	< 0.001 *	16 (8.2%)	1 (2.9%)	0 (0.0%)	8 (20.5%)	2 (20.0%)	< 0.05 *
**Comorbidities, assessed during baseline[8], n (%)**												
Cirrhosis	117 (60.3%)	90 (84.9%)	< 0.001 *	133 (60.7%)	72 (91.1%)	< 0.001 *	136 (69.7%)	18 (51.4%)	15 (71.4%)	30 (76.9%)	8 (80.0%)	0.17
Hypertension	106 (54.6%)	61 (57.5%)	0.72	119 (54.3%)	47 (59.5%)	0.51	96 (49.2%)	26 (74.3%)	11 (52.4%)	27 (69.2%)	7 (70.0%)	< 0.05 *
Diabetes mellitus	59 (30.4%)	54 (50.9%)	< 0.001 *	75 (34.2%)	38 (48.1%)	< 0.05 *	67 (34.4%)	13 (37.1%)	9 (42.9%)	21 (53.8%)	3 (30.0%)	0.22
Esophageal varices	27 (13.9%)	28 (26.4%)	< 0.05 *	30 (13.7%)	25 (31.6%)	< 0.001 *	38 (19.5%)	6 (17.1%)	2 (9.5%)	7 (17.9%)	2 (20.0%)	0.89
Ascites	14 (7.2%)	40 (37.7%)	< 0.001 *	16 (7.3%)	36 (45.6%)	< 0.001 *	34 (17.4%)	6 (17.1%)	5 (23.8%)	7 (17.9%)	2 (20.0%)	0.93
Encephalopathy	7 (3.6%)	15 (14.2%)	< 0.01 *	8 (3.7%)	13 (16.5%)	< 0.001 *	12 (6.2%)	2 (5.7%)	1 (4.8%)	6 (15.4%)	1 (10.0%)	0.30

Abbreviations – 1L: first-line; A+B: atezolizumab plus bevacizumab; ALBI: Albumin-bilirubin; BCLC: Barcelona Clinic Liver Cancer; CP: Child-Pugh; ECOG PS: Eastern Cooperative Oncology Group Performance Status; SD: standard deviation; uHCC: unresectable hepatocellular carcinoma.

Notes:

1. Statistical comparisons between subgroups were performed using chi-squared tests for categorical variables. Where expected counts ≤ 10, Fisher's exact tests were used instead of chi-squared tests. *P*-value < 0.05 was considered statistically significant and is denoted with an asterisk (*).
2. The Asian (non-Hispanic) subgroup included patients who were American Indian or Alaska Native, or Hawaiian.
3. Some patients' primary HCC etiology could only be established as viral/nonviral. As a result, subcategories may not sum to 100%.
4. Other primary HCC etiologies included alcohol abuse, autoimmune disease, autoimmune hepatitis, cirrhosis, cryptogenic non-viral etiology, hemochromatosis, hyperlipidemia, or hypertension.
5. Percentages may not add up to 100% because a small proportion of patients with unknown information has not been reported in the table.
6. Multiple responses were allowed for this question and percentages may add up to more than 100%.
7. ALBI grade was determined as follows: grade 1: ≤ -2.60; grade 2: > -2.60 and ≤ -1.39; grade 2a: > -2.60 and ≤ -2.118; grade 2b: > -2.118 and ≤ -1.39; grade 3: >-1.39.
8. The baseline period was defined as the period of up to 1 year prior to the initiation of A+B.

Supplementary Table S3. Use of EGD during the baseline period among patients with uHCC receiving A+B as 1L treatment.

	**Overall cohort N=300**
Patients with ≥1 EGD procedure during baseline[1]	224 (74.7%)
Timing of EGD procedure	
Less than 6 months prior to initiation of 1L A+B treatment	203 (90.6%)
6-12 months prior to initiation of 1L A+B treatment	22 (9.8%)
Reasons for EGD (among patients with ≥1 procedure)[2]	
Eligibility for treatment with A+B	126 (56.3%)
Standard workup for HCC	76 (33.9%)
Examine for potential bleeding	25 (11.2%)
Esophageal varices found (among patients with ≥1 procedure)	78 (34.8%)
Treatment prescribed for esophageal varices[2,3]	
No treatment	50 (64.1%)
Variceal band ligation	19 (24.4%)
Beta-blockers	8 (10.3%)

Abbreviations – 1L: first-line; A+B: atezolizumab plus bevacizumab; EGD: upper gastrointestinal esophagogastroduodenoscopy; HCC: hepatocellular carcinoma; uHCC: unresectable hepatocellular carcinoma.

Notes:

1. The baseline period was defined as the period of up to 1 year prior to the initiation of A+B.
2. Multiple responses were allowed for this question and percentages may add up to more than 100%.
3. Percentages may not add up to 100% because a small proportion of patients with unknown information has not been reported in the table.

Supplementary Table S4. HCC-related healthcare resource utilization in patients with uHCC receiving A+B as 1L treatment, during the first year of treatment with A+B.

	**Overall cohort N=300**
**HCC-related hospitalizations**	
Patients with ≥1 hospitalization	147 (49.0%)
Annualized number of hospitalizations (mean ± SD; PPPY)	2.9 ± 7.0
Average length of hospitalization per patient, among patients with ≥1 hospitalization (mean ± SD; days)	5.8 ± 4.7
Main reason for hospitalization among patients with ≥1 hospitalization[1]	
Symptom-related	79 (53.7%)
Disease progression	19 (12.9%)
Procedure/surgery	20 (13.6%)
Adverse events from treatments	13 (8.8%)
Treatment administration	3 (2.0%)
Other	43 (29.3%)
**HCC-related ER visits[2]**	
Patients with ≥1 ER visit	129 (43.0%)
Annualized number of ER visits (mean ± SD; PPPY)	2.2 ± 4.6
Main reason for ER visit among patients with ≥1 ER visit1	
Symptom-related	89 (69.0%)
Disease progression	8 (6.2%)
Adverse events from treatments	10 (7.8%)
Other	39 (30.2%)
**HCC-related oncology visits**	
Patients with ≥1 oncology visit	287 (95.7%)
Annualized number of oncology visits (mean ± SD; PPPY)	18.6 ± 30.1

Abbreviations – 1L: first-line; A+B: atezolizumab plus bevacizumab; ER: emergency room; HCC: hepatocellular carcinoma; PPPY: per patient per year; SD: standard deviation; uHCC: unresectable hepatocellular carcinoma.

Notes:

1. Multiple responses were allowed for this question and percentages may add up to more than 100%.
2. Percentages may not add up to 100% because a small proportion of patients with unknown information has not been reported in the table.
